# Prominin 2 decreases cisplatin sensitivity in non-small cell lung cancer and is modulated by CTCC binding factor

**DOI:** 10.2478/raon-2023-0033

**Published:** 2023-09-04

**Authors:** Jiyang Tang, Dejun Shu, Zhimin Fang, Gaolan Yang

**Affiliations:** Department of Thoracic Surgery, The Third Affiliated Hospital of ZunYi Medical University (The First People's Hospital of ZunYi), Zunyi, Guizhou, China

**Keywords:** non-small cell lung cancer, PROM2, cisplatin, drug resistance, CTCF

## Abstract

**Background:**

Non-small cell lung cancer (NSCLC) is the major pathological type of lung cancer and accounts for the majority of lung cancer-related deaths worldwide. We investigated the molecular mechanism of prominin 2 (PROM2) involved in cisplatin resistance in NSCLC.

**Patients and methods:**

The GEO database was analyzed to obtain differential genes to target PROM2. Immunohistochemistry and western blotting were used to detect protein expression levels. To examine the role of PROM2 in NSCLC, we overexpressed or knocked down PROM2 by transfection of plasmid or small interfering RNA. In functional experiments, CCK8 was used to detect cell viability. Cell migration and invasion and apoptosis were detected by transwell assay and flow cytometry, respectively. Mechanistically, the regulation of PROM2 by CTCF was detected by ChIP-PCR. *In vivo* experiments confirmed the role of PROM2 in NSCLC.

**Results:**

GEO data analysis revealed that PROM2 was up-regulated in NSCLC, but its role in NSCLC remains unclear. Our clinical samples confirmed that the expression of PROM2 was markedly increased in NSCLC tissue. Functionally, Overexpression of PROM2 promotes cell proliferation, migration and invasion, and cisplatin resistance. CTCF up-regulates PROM2 expression by binding to its promoter region. *In vivo* experiments confirmed that PROM2 knockdown could inhibit tumor growth and increase the sensitivity of tumor cells to cisplatin.

**Conclusions:**

PROM2 up-regulation in NSCLC can attenuate the sensitivity of NSCLC cells to cisplatin and promote the proliferation, migration and invasion of tumor cells. PROM2 may provide a new target for the treatment of NSCLC.

## Introduction

Non-small cell lung cancer (NSCLC), consisting of adenocarcinoma and squamous cell carcinoma, is the major pathological type of lung cancer, accounting for the majority of lung cancer-related deaths worldwide.^1,2^ Despite advances in the diagnosis and treatment of patients with NSCLC, majority of the patients are diagnosed with advanced metastasis or recurrence, resulting in poor overall 5-year survival rates of patients with NSCLC.^3^ So far, platinum and its derivatives are still the main choice for anticancer chemotherapy.^4^ However, platinum-based chemotherapy drugs resistance is often developed during lung cancer treatment.^5^ Since drug resistance mechanism is only limitedly investigated, the exact mechanisms underlying cisplatin resistance in NSCLC remain to be determined. Thus, a deeper understanding of the mechanism of cisplatin resistance will provide new ideas for discovering potential therapeutic targets and promoting therapeutic efficacy in clinic.

Prominin 2 (PROM2) is an important member of the pentaspan transmembrane family and is enriched at plasma membrane protrusions.^6^ PROM2 has recently been shown to have an anti-ferroptosis effect. The expression of PROM2 can be rapidly induced by stimulants that increase lipid peroxidation and promote the formation of a multi-vesicular body (MVB) containing ferritin. These MVBs export as exosomes to reduce intracellular iron concentration, thereby alleviating cell ferroptosis.^7,8^ PROM2 is activated by p38-mediated HSF1 transcription to antaonized 4HNE or RSL3-induced ferroptosis.^9^ Notably, PROM2 can promote gemcitabine resistance by activating Akt signaling pathway in pancreatic cancer.^10^ However, the role and mechanism of PROM2 in NSCLC remains unclear.

CTCC binding factor (CTCF) is a transcription factor with 11 zinc fingers that is highly conserved despite being over 700 amino acids in length.^11^ As a multifunctional transcription factor, it has been reported that CTCF is involved in the occurrence of multiple cancers.^12,13^ CTCF promotes colorectal cancer cell proliferation and chemotherapy resistance to 5-FU by targeting p53-hedgehog axis.^14^ Notably, CTCF promotes the progression of head and neck squamous cell carcinoma and drug resistance to cisplatin and 5-FU by targeting HOXA9.^15^ Nevertheless, the role of CTCF in NSCLC is extremely limited.

In this study, our results showed that PROM2 was up-regulated in NSCLC *in vivo* and *in vitro*. More importantly, ENCODE ChIP-seq data predicted the binding between CTCF and PROM2 promoter. Subsequently, the CTCF/PROM2 modulated the NSCLC cell proliferation, migration and invasion, and cisplatin resistance.

## Patients and methods

The GEO microarray data GSE32863 (https://www.ncbi.nlm.nih.gov/geo/query/acc.cgi?acc=GSE32863) was analyzed to compare the expression of differential genes (DEGs) in NSCLC tissues and adjacent normal tissues. The expression level of PROM2 in lung adenocarcinoma (TCGA-LUAD) and lung squamous cell carcinoma (TCGA-LUSC) was analyzed by online platform GEPIA based on TCGA database (http://gepia.cancer-pku.cn/detail.php?gene=PROM2). Kaplan-Meier plotter was used to analyze the relationship between PROM2 expression and prognosis according to the TCGA database (https://portal.gdc.cancer.gov/). To explore the upstream regulation of PROM2, ENCODE ChIP-seq data were performed to predict the binding between PROM2 promoter and CTCF. In addition, the CTCF expression and the correlation between CTCF and PROM2 were assessed by TIMER 2.0 analysis and GEPIA platform analysis.

### Clinical samples

Our study has been authorized by the Ethics Committee of the Third Affiliated Hospital of ZunYi Medical University (The First People's Hospital of ZunYi). A total of 35 NSCLC and adjacent non-tumor tissues were collected and stored in −80°C. The patient characteristics was listed in Table 2. All procedures performed in studies involving human participants were in accordance with the standards upheld by the Ethics Committee of the Third Affiliated Hospital of ZunYi Medical University (The First People's Hospital of ZunYi) and with those of the 1964 Helsinki Declaration and its later amendments for ethical research involving human subjects.

All animal experiments were approved by the Ethics Committee of the Third Affiliated Hospital of ZunYi Medical University (The First People's Hospital of ZunYi) for the use of animals and conducted in accordance with the National Institutes of Health Laboratory Animal Care and Use Guidelines.

The animal experiment complies with the ARRIVE guidelines and in accordance with the National Institutes of Health guide for the care and use of Laboratory animals (NIH Publications No. 8023, revised 1978).

### Cell culture and transfection

Human normal lung epithelial cells (BEAS-2B) and lung cancer cells (NCI-H1650, A549, NCI-H1299, PC-9) were purchased from American Type Culture Collection (ATCC, MA, VA, USA), cultured in RPMI-1640 supplemented with 10% fetal bovine serum (FBS; Gibco, Grand Island, USA) and incubated at 37°C in 5% CO_2_.

For transfection, the A549 and PC-9 cells were transfected with transfecting plasmid or Lentivirus to overexpress or knock down PROM2 using Lipofectamine®3000 (In vitrogen, Carlsbad, CA, USA) reagent.^16^ For co-transfection, the A549/DPP cells were co-transfected with small interfering RNA of CTCF and/or plasmid of PROM2, and then the cell viability and proliferation were examined.

### To construct cisplatin-resistant lung cancer cell lines

Resistant NSCLC cells were established by continuously exposing A549 and PC-9 cells to cisplatin in a series of concentration gradients (0.1 μM to 6 μM). Cells that survived in cell medium with 6 μM cisplatin were identified as cisplatin resistant cells (A549/DDP, PC-9/DDP). Thereafter, the parental cells or drug-resistant cells were treated with different concentrations of cisplatin (0, 1.0 μM, 10 μM, 50 μM, 100 μM, 200 μM). CCK-8 was performed to measure cell viability and calculated half maximal inhibitory concentration (IC_50_).

### Immunohistochemistry

The tissues were fixed in 4% paraformaldehyde, embedded with paraffin, sectioned at 4 μm, dewaxed in xylene, soaked in 3% hydrogen peroxide solution to eliminate endogenous catalase, and repaired in citrate solution pH 6.0 at high temperature. Primary antibody PROM2 (Abcam, Cambridge, UK; ab118492; 1:100) was added after serum blocking. After overnight at 4°C, the sections were developed by DAB, counterstained with hematoxylin for observation.

### Western blotting

Clinical tissue samples or cells of each group were collected, and RIPA lysis buffer was added to extract total protein. Appropriate amounts of protein were subjected to sodium dodecyl sulfate-polyacrylamide gel electrophoresis. After electrophoresis, the protein was transferred to PVDF membrane, blocked in 5% skim milk for 1 h at 25°C, added with primary antibody, and incubated overnight at 4°C on a shaker.^17^ Specific primary antibodies are as follows: PROM2 (Abcam, ab74997, 1:1000), CTCF (Abcam, ab128873), β-actin (Abcam, ab8226). After incubation with secondary antibodies, electrochemical luminescence reagent was added without light. Image J software was used to analyze the gray value of the strips.

### CCK8 assay

5 × 10^3^ cells/mL cells were seeded into a 96-well plate (100 μl/well) and then cultured for 0, 1, 2 and 3 days, respectively. CCK-8 solution (Beyotime, Shanghai, China; 10 μl) was added to each well, and the culture was continued for 2 h.^18^ The absorbance value (OD value) at 450 nm was detected by microplate reader.

### Clone formation

The cells after transfection were seeded in 6-well plates and cultured for 14 days. When visible clones appeared, the colonies were stained with gentian violet (Goodbio Technology, Wuhan, China) for 30 min. The proliferation of cells was observed under a microscope.

### Transwell assay

For cell migration assay, 100 μl cell suspension was added to the upper chamber, and 600 μl RPMI-1640 containing 10% FBS was added to the lower chamber. The cells were cultured for 24 h at 37°C in a 5% CO_2_ incubator. For cell invasion assays, Transwell chambers coated with extracellular matrix gel were used, and the rest of the procedure was the same as for cell migration assays. At the end of culture, the upper chamber was removed and the cells on the inner surface of the filtration membrane of the chamber were wiped off. The cells were fixed with 4% paraformaldehyde, stained with 0.1% crystal violet, observed by microscope, counted and photographed. Five fields of view were randomly selected and averaged.

### Flow cytometry

Cells (2 × 10^6^ cells/mL) were seeded into 96-well cell culture plate and incubated at room temperature for 24, 48 and 72 h. The cells were treated according to Annexin V-FITC/PI apoptosis kit (Beyotime) instructions. The cells were resuspended in 300 μl PBS. The cells were stained with Annexin V (5 μl) and PI (5 μl) for 15 min at room temperature, and the apoptosis rate was detected by flow cytometry within 4 h.^19^

### Chromatin Immunoprecipitation-quantitative real-time PCR (ChIP-PCR)

A549 cells were transfected with pcDNA-CTCF or CTCF small interfering RNA and cultured in an incubator containing 5% CO_2_ at 37°C for 24 h. After removal of the medium, the cells were fixed with 16% paraformaldehyde. They were divided into IgG+siNC group, CTCF+siNC group, IgG+siCTCF group and CTCF+siCTCF group. CTCF-bound DNA was captured using antibodies according to the ChIP kit (Cell signaling Technology, Boston, MA, USA) instructions. PCR was used to verify the capture of CTCF gene promoter DNA, and ChIP-PCR was used for quantitative analysis. Immunoprecipitation efficiency was calculated using input sample percentage method.

### *In vivo* experiment

Our study was approved by the Animal Ethics Committee of the Third Affiliated Hospital of ZunYi Medical University (The First People's Hospital of ZunYi). BALB/c nude mice were subcutaneously injected with PROM2 knockdown stable A549/DDP cells (5 × 10^6^). One week later, cisplatin (4 mg/kg) was injected into the peritoneum every 3 days. All nude mice were divided into 5 groups: control group, shNC group, cisplatin +shNC group, shPROM2#1 group and cisplatin +shPROM2#1 group, with 5 mice in each group. On the 25th day, the nude mice were sacrificed under anesthesia. The weight of the tumors was weighed and the volume of the tumors was measured. The tumors were collected for subsequent experiments.

### Statistical analysis

Graphpad 7.0 software was used for data analysis. Data were expressed as mean ± standard deviation, and comparison between two groups was performed by *t* test. One-way analysis of variance analysis of variance was used to compare the differences between more than two groups. *P* < 0.05 was considered statistically significant. IC50 value was calculated by Graphpad according to the results of CCK-8 assay.

## Results

### PROM2 is up-regulated in NSCLC

**FIGURE 1.**
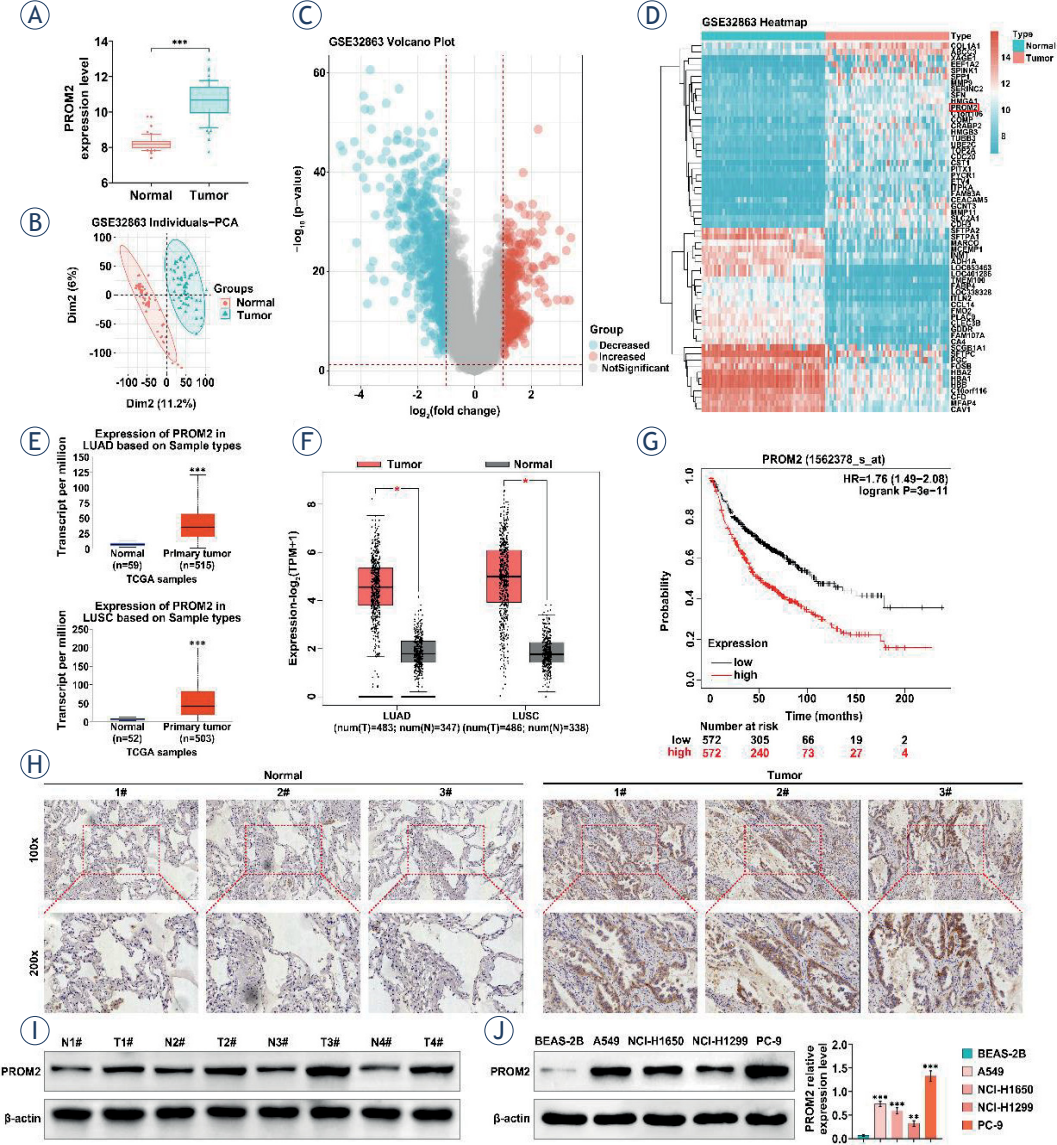
PROM2 is overexpressed in non-small cell lung cancer (NSCLC). **(A)** Expression level of PROM2 in normal group and tumor. **(B–D)** GEO microarray data GSE32863 was analyzed by LIMMA package using R language to compare the expression of differential genes (DEGs) in lung cancer tissues and adjacent normal tissues. The data were corrected and analyzed by PCA **(B)**, and the volcano map **(C)** and heat map **(D)** were drawn. **(E–F)** To analyze the expression level of PROM2 in non-small cell lung cancer (TCGA-LUAD and TCGA-LUSC) using online platform GEPIA based on TCGA database. **(G)** Kaplan-Meier plotter was used to analyze the effect of PROM2 expression on prognosis. **(H)** Representative immunohistochemical picture of PROM2 in NSCLC. **(I)** PROM2 protein level in NSCLC by Western blotting. **(J)** The protein expression of PROM2 in human normal lung epithelial cells (BEAS-2B) and lung cancer cells (NCI-H1650, A549, NCI-H1299, PC-9) was detected by western blotting. ^*^P < 0.05, ^**^P < 0.01, ^***^P < 0.001 compared with normal group/BEAS-2B group

To explore the pathogenesis of NSCLC, we analyzed the data GEO database data (GSE32863) confirmed to compare the expression of differential genes in NSCLC tissues and adjacent normal tissues and the results showed that PROM2 was up-regulated in NSCLC compared to adjacent normal tissues (Figure 1A). Interestingly, GEO microarray data GSE32863 confirmed the differential genes (DEGs) in lung cancer tissues and adjacent normal tissues. Notably, PROM2 expression was increased in lung cancer tissues (Figure 1B–1D). More importantly, online platform GEPIA data showed that both PROM2 transcript and expression levels were promoted in LUSC and LUAD patients compared to normal subjects (Figure 1E and 1F). Similarly, high expression of PROM2 predicted poor prognosis (Figure 1G). Representative images of IHC confirmed that PROM2 was highly expressed in NLCSC patient tissues compared to adjacent tissues (Figure 1H). Consistently, PROM2 expression was significantly enhanced in lung cancer cells (NCI-H1650, A549, NCI-H1299, PC-9) compared to BEAS-2B, especially in PC-9 and A549 cells (Figure 1J). Thus, the PC-9 and A549 cells was selected for subsequent experiments. These results indicated that the expression levels of PROM2 were up-regulated in NSCLC.

### PROM2 promotes the proliferation of lung cancer cells

**FIGURE 2.**
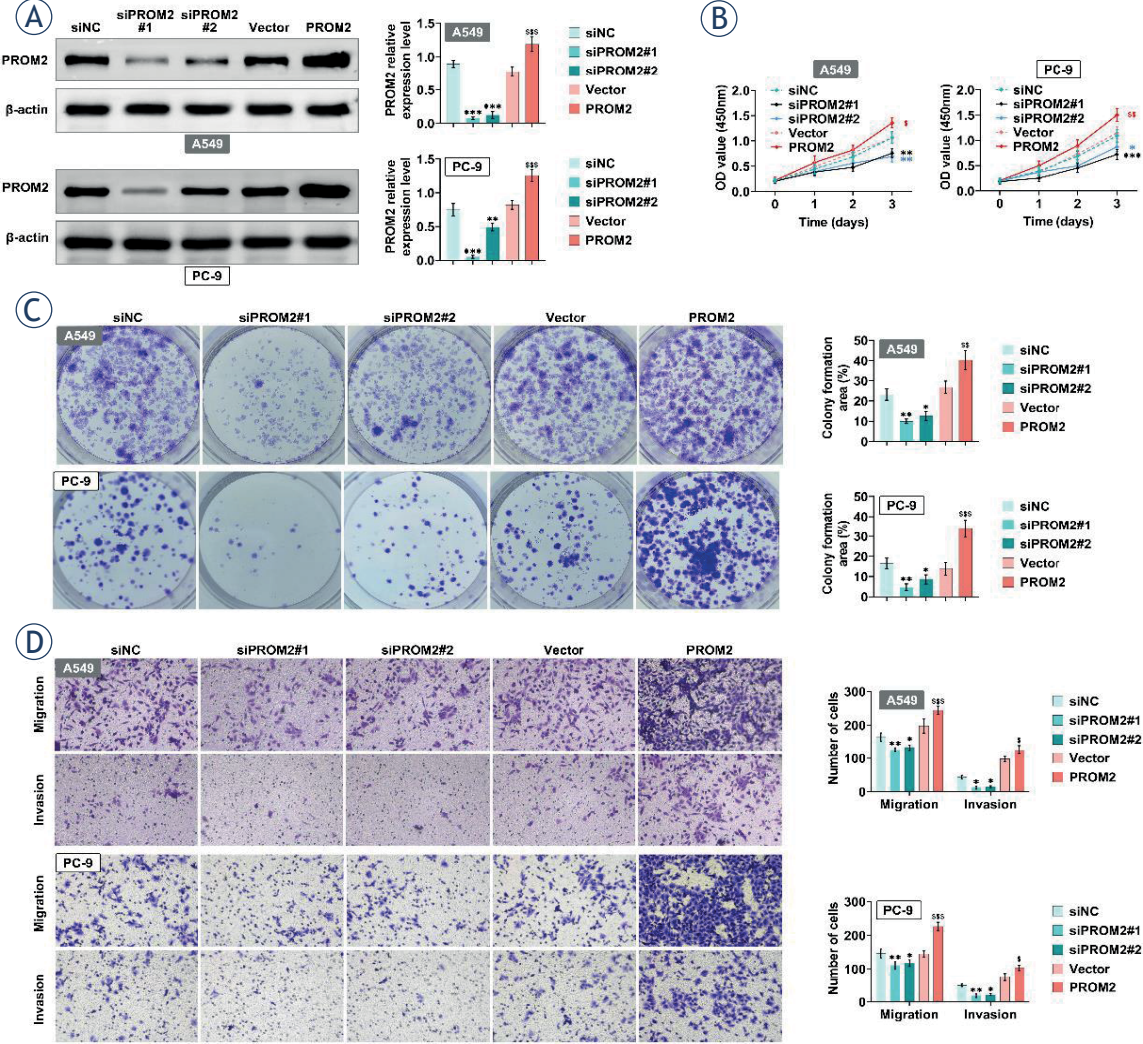
PROM2 promotes the proliferation of lung cancer cells. **(A–D)** PROM2 was overexpressed or knocked down in A549 and PC-9 cells. The protein level of PROM2 was detected by western blotting **(A)**, cell viability was detected by CCK8 **(B)**, cell proliferation was detected by clonal formation **(C)**. Transwell was used to detect cell migration and cell invasion **(D)**. ^*^P < 0.05, ^**^P < 0.01, ^***^P < 0.001 compared with siNC; ^$^*P* < 0.05, ^$$^*P* < 0.01, ^$$$^*P* < 0.001 compared with vector

To explore the role of PROM2 in NSCLC, PROM2 was overexpressed or knocked down in A549 and PC-9 cells. As expected, PROM2 was efficiently over-expressed or knocked down (Figure 2A). Then, the effects of altered PROM2 on NSCLC viability and motility were examined. As shown in Figure 2B, A549 and PC-9 cell viability was remarkably increased after PROM2 overexpression, and was decreased by knockdown of PROM2. In addition, knockdown of PROM2 reduced the number of colonies, whereas overexpression of PROM2 exhibited opposite effect (Figure 2C). Furthermore, knockdown of PROM2 observably inhibited the number of migrated and invaded cells, while over-expression of PROM2 increased the number of migrated and invaded cells (Figure 2D). These findings indicated that PROM2 can promote the cell viability, proliferation, migration and invasion of lung cancer cells.

### Effect of PROM2 on cisplatin sensitivity in lung cancer cells

**FIGURE 3.**
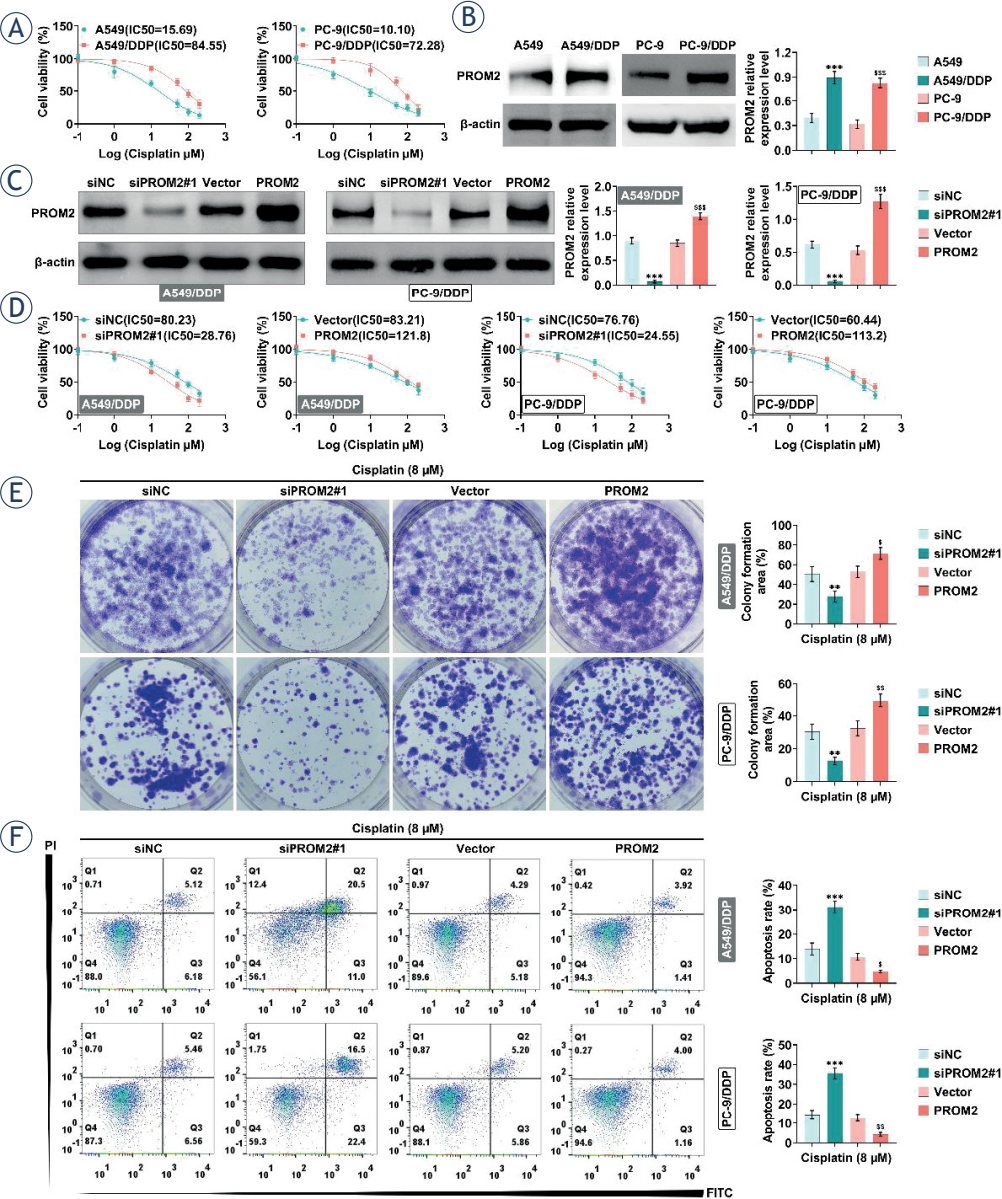
PROM2 attenuates the sensitivity of lung cancer cells to cisplatin. **(A)** Cell viability was detected by CCK8. **(B)** The expression level of PROM2 in different groups of cells was detected by western blotting. **(C–F)** PROM2 was knocked down or overexpressed in A549/DDP and PC-9/DDP, and then the protein level of PROM2 was detected by western blotting, cell viability was detected by CCK8 **(D)**, cell proliferation was detected by clone formation **(E)**, and cell apoptosis was detected by flow cytometry **(F)**. ^**^P < 0.01, ^***^P < 0.001 compared with A549 group; ^$^*P* < 0.05, ^$$^*P* < 0.01, ^$$$^*P* < 0.001 compared with vector

Subsequently, to investigate the effect of PROM2 on cisplatin sensitivity in NSCLC, we constructed drug-resistant cell lines (A549/DDP and PC-9/DDP). CCK8 assay results showed that both A549/DDP and PC-9/DDP cells had a lower cisplatin sensitivity than A549 (IC_50_ 84.55 *vs*. 15.69) and PC-9 cells (IC_50_ 72.28 *vs*. 10.10) (Figure 3A). Moreover, PROM2 expression level was significantly higher in resistant cells than in parental cells (Figure 3B). To investigate the role of PROM2 in drug-resistant cells, PROM2 was down-regulated or over-expressed in A549/DDP and PC-9/DDP cells (Figure 3C). Subsequently, CCK-8 assay implied that knockdown of PROM2 enhanced cisplatin sensitivity in A549/DPP (IC_50_ 28.76 *vs*. 79.28) and PC-9/DDP (IC_50_ 27.02 *vs*. 70.43) cells, while over-expression of PROM2 inhibited cisplatin sensitivity in A549/DPP (IC_50_ 112.40 *vs*. 81.80) and PC-9/DDP (IC_50_ 98.37 *vs*. 67.66) cells (Figure 3D). Moreover, the colony formation results indicated that knockdown of PROM2 reduced cell proliferation but overexpression of PROM2 promoted cell proliferation of A549/DPP and PC-9/DDP cells (Figure 3E). Consistently, flow cytometry analysis manifested that downregulation of PROM2 increased apoptosis, while overexpression of PROM2 decreased apoptosis of A549/DPP and PC-9/DDP cells (Figure 3F). These results unfolded that PROM2 reduced cisplatin sensitivity through reducing apoptosis and promoting proliferation.

### Knockdown of PROM2 enhances the sensitivity of lung cancer cells to cisplatin *in vivo*

**FIGURE 4.**
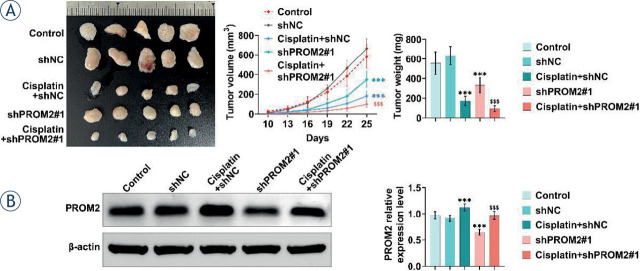
PROM2 enhances cisplatin resistance in lung cancer cells *in vivo*. BALB/c nude mice were subcutaneously injected with PROM2 knockdown stable A549/DDP cells. One week later, cisplatin (4 mg/kg) was injected into the peritoneum every 3 days. After 30 days, the cells were removed and the volume and weight were measured. **(A)** The volume and weight of tumors in different groups were detected. **(B)** The protein levels of PROM2 in different groups were detected by Western blotting. ^***^P < 0.001 compared with shNC group; ^$^*P* < 0.05, ^$$^*P* < 0.01, ^$$$^*P* < 0.001 compared with cisplatin+shNC

BALB/c nude mice were subcutaneously injected with PROM2-knockdown A549/DDP cells to investigate the effect of PROM2 *in vivo*. The data indicated that cisplatin treatment reduced the volume and weight of tumors, while knockdown of PROM2 further inhibited the tumor growth (Figure 4A). More importantly, cisplatin treatment increased PROM2 expression, which was decreased by shPROM2 (Figure 4B). All these results suggested that PROM2 knockdown suppressed tumor growth through enhancing the cisplatin sensitivity.

### CTCF up-regulates PROM2 expression by binding to its promoter region

**FIGURE 5.**
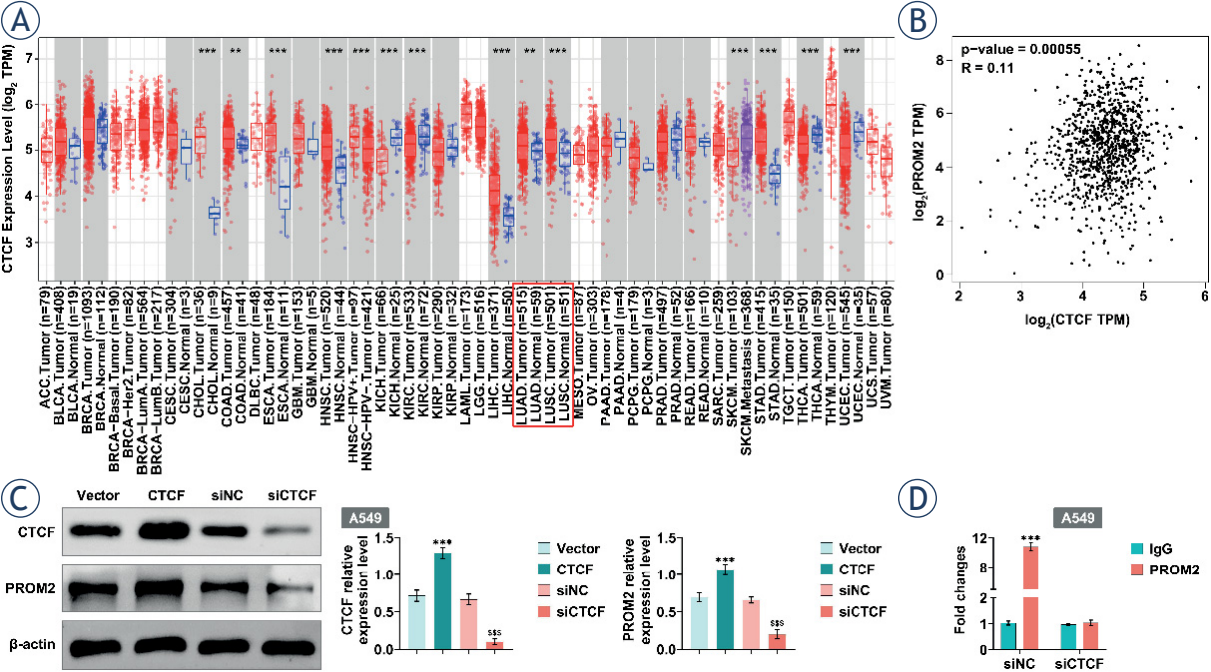
Up-regulation of PROM2 induced by CTCF. **(A)** TIMER 2.0 data analysis revealed that CTCF was overexpressed in lung cancer. **(B)** There was a positive correlation between CTCF and PROM2 in LUAD and LUSC data analyzed by GEPIA platform. **(C)** Protein levels of PROM2 and CTCF were detected by western blotting. **(D)** The expression level of PROM2 was detected by ChIP-PCR. ^**^P < 0.01, ^***^P < 0.001 compared with vector group; ^$$$^*P* < 0.001 compared with siNC

By analyzing Chip ChIP-seq data, it was found that CTCF, REST, MAFK, and TEAD4 can bind to PROM2 promoter, especially CTCF (Table 1). In addition, TIMER 2.0 data showed that CTCF was overexpressed in lung cancer (Figure 5A). Besides, GEPIA platform analysis revealed a positive correlation between CTCF and PROM2 in LUAD and LUSC (Figure 5B). Furthermore, overexpression of CTCF significantly promoted PROM2 expression, while knockdown of CTCF reduced PROM2 expression in A549 cells (Figure 5C). Interestingly, ChIP-PCR result unfolded that the CTCF distinctly bound to PROM2 promoter (Figure 5D). These findings concluded that CTCF promoted PROM2 expression via directly binding to its promoter.

**TABLE 1.** Data of ENCODE ChIP-seq

**TFs**	**Signal peak**	**ENCODE ID**
CTCF	114.601	ENCFF797HKW
REST	94.922	ENCFF044DWW
MAFK	71.984	ENCFF757FDG
TEAD4	34.118	ENCFF186WSI

ChIP-seq = transcription factor chromatin immunoprecipitation-DNA sequencing; CTCF = transcriptional repressor 11-zinc finger protein; MAFK = bZip Maf transcription factor protein; REST = neuron-restrictive silencer factor; TEAD4 = member of the transcriptional enhancer factor family; TFs = transcription factors

**TABLE 2.** The patient characteristics had no statistical significance

**Clinicopathological factor**	**Number of cases**	**PROM2 expression**		**P value**

		**High**	**Low**	
Total Cases	35	19	16	
Gender				0.606
Male	22	13	9	
Female	13	6	7	
Age				0.814
< 60	17	9	8	
≥ 60	18	10	8	
Histological type				0.189
LSCC	12	9	3	
LAD	15	7	8	
LCLC	8	3	5	
Pathological grading				0.002
I	15	3	12	
II	11	8	3	
III	9	8	1	
TNM stage				0.006
I	13	3	10	
II	12	7	5	
III	10	9	1	
Smoking history				0.3320
yes	24	13	11	
no	11	6	5	

LAD = lung adenocarcinoma; LCLC = non-small cell lung cancer no other specified; LSCC = squamous cell lung carcinoma

### Knockdown of CTCF can increase the sensitivity of lung cancer cells to cisplatin by down-regulating PROM2

**FIGURE 6.**
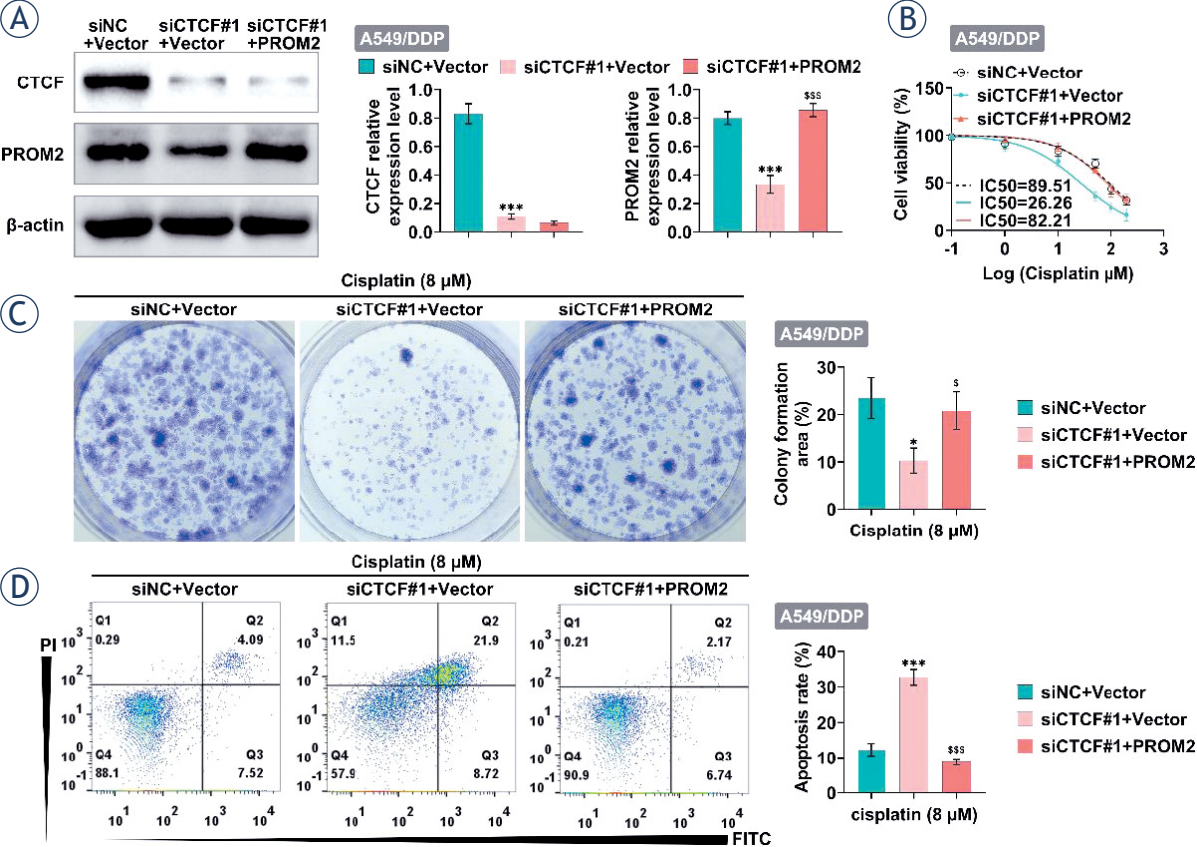
CTCF knockdown increased the sensitivity of lung cancer cells to cisplatin by down-regulating PROM2. **(A–D)** After knockdown of CTCF and/or overexpression of PROM2 in A549/DDP, the protein levels of CTCF and PROM2 were detected by western blotting **(A)**, cell viability was detected by CCK8 **(B)**, cell proliferation was detected by clone formation **(C)**, and cell apoptosis was detected by flow cytometry **(D)**. ^*^P < 0.05, ^***^P < 0.001 compared with siNC+vector; ^$^*P* < 0.05, ^$$$^*P* < 0.001 compared with siCTCF#1+vector

To explore the effect of CTCF on cisplatin sensitivity in NSCLC, CTCF was knocked down in A549/DDP cells. As shown in Figure 6A, siCTCF decreased PROM2 expression, which was increased by overexpression of PROM2. Moreover, CCK-8 assay showed that siCTCF promoted cisplatin sensitivity (IC_50_ 29.26 *vs*. 89.51), while overexpression of PROM2 attenuated this effect (Figure 6B). Furthermore, knockdown of CTCF reduced the number of colony formation of A549/DDP cells, which was reversed by overexpression of PROM2 (Figure 6C). Consistently, the apoptosis rate was increased by knockdown of CTCF in A549/DDP cells, which was decreased by PROM2 overexpressed (Figure 6D). Thus, these findings revealed that knockdown of CTCF can increase the sensitivity of lung cancer cells to cisplatin through down-regulating PROM2.

## Discussion

Lung cancer is the most common cancer and the leading cause of cancer-related death worldwide, with NSCLC accounting for about 80% of all lung cancers in the United States.^20,21^ We analyzed the database and found that PROM2 was highly expressed in NSCLC and associated with poor prognosis. More importantly, we demonstrated that PROM2 promoted the proliferation, migration and invasion of lung cancer cells, and inhibited the apoptosis, and their sensitivity to cisplatin *in vitro*. *In vivo* experiments confirmed that knockdown of PROM2 enhances the sensitivity of lung cancer cells to cisplatin, thereby inhibiting tumor growth. Furthermore, CTCF up-regulated PROM2 expression and reduced cisplatin sensitivity through directly binding to PROM2 promoter, providing a novel sight for the mechanism of the cisplatin resistance in NLCSC.

Previous studies have found that PROM2 was up-regulated in a variety of tumors, such as bladder cancer, pancreatic cancer, melanoma.^7,10,22^ However, the potential role and mechanism of PROM2 in NSCLC remains unclear. In this study, the expression of PROM2 was up-regulated in NSCLC, indicating that PRMO2 was involved in the pathogenesis of NSCLC. Consistently, our clinical samples confirmed that the protein level of PROM2 was significantly increased in NSCLC tissues. Cell proliferation, migration and invasion are responsible for tumorigenesis and poor prognosis.^23^ Our study found that overexpression of PROM2 promoted the proliferation, migration and invasion of lung cancer cells, which might be the first time exploring the carcinogenesis role of PROM2 in NSCLC. Fortunately, a recent report has pointed out that activated PROM2 serves as a tumorigenic regulator in bladder cancer via attenuating ferroptosis.^7^

Cisplatin-based chemotherapy remains the standard care for NSCLC patients, but many patients are prone to develop drug resistance after cisplatin treatment.^24^ It has been reported that many RNAs and proteins participate in modulating cisplatin resistance in NSCLC patients.^25,26^ Recently, Li *et al*. have demonstrated that PROM2 promotes gemcitabine chemoresistance via activating the Akt signaling pathway in pancreatic cancer.^10^ In this study, through constructing drug-resistant cell lines (A549/DDP and PC-9/DDP), we found that PROM2 reduced cisplatin sensitivity in lung cancer cells. Besides, reporters have confirmed that cisplatin resistance in NSCLC can result from alterations in regulation of the cell apoptosis.^27^ Based on the results that high expression of PROM2 promoted the proliferation, migration and invasion, we hypothesized that PROM2 can decrease cisplatin sensitivity via enhancing NSCLC cells survival and metastasis, thereby promoting cisplatin resistance.

DNA-binding proteins can modulate proteins expression via binding to its promoter. To confirm the regulatory mechanism of PROM2 expression, CTCF was predicted to bind to PROM2 promoter. Interestingly, most gained CTCF binding events exhibit enhancer activities and are induced by oncogenic transcription factors.^28^ Consistently, we demonstrated that CTCF could bind to PROM2 promoter and up-regulate PROM2 expression. Mechanically, cisplatin induces dormant and reactivated lung cancer cells, and CTCF governs the entry of cancer cells into dormant states and control the re-entry of dormant cancer cells into the cell cycle.^29^ Thus, we suspected that CTCF up-regulates PROM2 expression and governs the shift of cellular dormancy and reactivation under cisplatin stimulation, subsequently promotes cell proliferation and inhibits apoptosis, thereby reducing the cisplatin sensitivity. However, the relationship between cisplatin resistance and migration phenotype is still unknown. It has been reported that the increases in cell invasion and migration abilities may be a consequence of cisplatin resistance, resulting in enhanced cancer metastasis after long-term treatment with cisplatin.^30^ Therefore, we suspected that up-regulation of CTCF/PROM2 decreases cisplatin sensitivity and then enhances NSCLC cell migration and invasion. There are still some limitations in our study, and we have yet to show whether CTCF/PROM2 mechanism is the only mechanism of enhancement of cisplatin resistance in NSCLC. Other therapeutic targets of mechanism of cisplatin resistance are still unknown.

## Conclusions

In conclusion, our study found that PROM2 was up-regulated in NSCLC and promoted NSCLC cells proliferation, invasion and migration, as well as the drug resistance of lung cancer cells to cisplatin, providing a theoretical target for the treatment of NSCLC, and a novel sight for therapeutic strategy for NSCLC.
